# Creation of a completely helper cell-dependent recombinant morbillivirus

**DOI:** 10.1099/vir.0.050872-0

**Published:** 2013-06

**Authors:** Jana Baron, Michael Baron

**Affiliations:** The Pirbright Institute, Ash Road, Pirbright, Surrey GU24 0NF, UK

## Abstract

We have created a completely helper cell-dependent morbillivirus by modifying the genome to remove the coding sequence of the phosphoprotein (P) and recovering the recombinant virus in a cell line constitutively expressing the P protein. The P protein-deleted virus (P^−^) grew very inefficiently unless both of the viral accessory proteins (V and C) were also expressed. Growth of the virus was restricted to the P-expressing cell line. The P^−^ virus grew more slowly than the parental virus and expressed much less viral protein in infected cells. The technique could be used to create virus-like particles for use as a vaccine or as antigen in immunological or serological assays.

The genus *Morbillivirus* includes a number of important human (*Measles virus*, MeV), livestock (*Rinderpest virus*, RPV; *Peste-des-petits-ruminants virus*, PPRV) or other animal (*Canine distemper virus*) pathogens. In some cases, growth or handling of the live viruses requires very high level containment, which can lead to problems and restrictions when preparing material where correctly folded surface glycoproteins are required, for example for use in serological ELISAs, and the glycoproteins are only folded properly in the virus, or a virus-like particle of some kind. We have sought to establish a system by which the virus growth could become completely dependent on a helper cell line, thereby making the production, shipment and use of the resultant virus entirely biosafe. One possible way that this might be done is by removing the coding sequence for an essential viral protein and providing that protein *in trans* in a modified cell line. We show here that this is possible for this group of viruses.

Morbilliviruses, like other paramyxoviruses, are non-segmented negative-strand RNA viruses. They have six genes (or transcription units), encoding, respectively, the nucleocapsid protein (N), phosphoprotein (P), matrix protein (M), the two envelope glycoproteins (F and H) and the viral RNA polymerase (L). The P gene also gives rise to two non-structural or accessory proteins, V and C, which play a variety of roles in modulating host immune responses and the dynamics of virus replication (e.g. Baron & Barrett, 2000; Nakatsu *et al.*, 2008; Nanda & Baron, 2006; Ohno *et al.*, 2004; Palosaari *et al.*, 2003; Parks *et al.*, 2006; Takeuchi *et al.*, 2003; Tober *et al.*, 1998), but are not essential proteins in the virus life cycle, since recombinant morbilliviruses lacking either or both proteins have been made which grow in cell culture (Baron & Barrett, 2000; Radecke & Billeter, 1996; Schneider *et al.*, 1997). When selecting a viral gene to delete, we took into consideration that recombinant forms of MeV lacking their M protein have been made which can grow in cell culture, albeit very inefficiently (Cathomen *et al.*, 1998). Removing either one of the glycoproteins would not prevent viral replication, only limit its spread. The P protein was selected as the viral protein to provide *in trans* as it acts as a subunit of the polymerase, as well as working with the N protein in encapsidation of the genome, and is thus completely indispensable in the virus life cycle. In addition, the interactions of P with N and L have been found to be largely virus specific (Brown *et al.*, 2005), meaning that the efficient replication of a virus depends on the presence of its own P protein. Recombination has not been observed naturally in the order *Mononegavirales*, so there is no way for the virus to recover a deleted gene, even if it co-infected a cell along with a wild-type version of the virus.

Methods for making recombinant versions of morbilliviruses have been established for some time (Baron & Barrett, 1997; Gassen *et al.*, 2000; Radecke *et al.*, 1995), although the rescue of recombinant PPRV was only recently achieved (Hu *et al.*, 2012). Since our initial aim was to prepare virus-like material with the surface glycoproteins of PPRV for use in a competition ELISA that is used to screen for anti-PPRV antibodies (Anderson & McKay, 1994), and since there was no working system to make recombinant PPRV available at the time we initiated this project, we used an existing chimeric virus in which the outer structure (M, F and H proteins) are from PPRV while the core replication machinery (N, P and L) and the promoters are from RPV, RPV_PPRMFH (Mahapatra *et al.*, 2006). The genome of RPV_PPRMFH was modified to either completely remove the P gene (RPV_PPRMFH-P^−^) or replace the normal P gene with one encoding either the C protein alone (RPV_PPRMFH-P^−^C^+^) or both the C and V proteins (RPV_PPRMFH-P^−^VC^+^) (Fig. 1a). In the case of the P^−^VC^+^ construct, the editing site in the P/V shared sequence was altered, as previously described (Baron & Barrett, 2000), to prevent the co-transcriptional editing that normally gives rise to both P and V proteins from the same gene (Cattaneo *et al.*, 1989) and an extra G inserted at the editing site to give a V-type message. We based the helper cell line on Vero cells expressing the canine form of SLAM (signalling lymphocyte activation molecule, the universal morbillivirus receptor; Tatsuo & Yanagi, 2002); this cell line, Vero-Dog-SLAM (VDS) (von Messling *et al.*, 2003), has been particularly good in our hands at supporting the replication of PPRV. The coding sequence of the P protein was modified to prevent translation of the overlapping C-protein reading frame, but without changing the sequence of the resultant P protein; since transcription in the host cell is by the cellular RNA polymerase II, rather than the viral RNA polymerase, editing does not occur and the V protein is not made. This P coding sequence was placed into pcDNA6 (Life Technologies), which carries a selectable blasticidin resistance marker. The resultant plasmid was transfected into VDS cells and a P protein-expressing clonal cell line (VDS-P) selected by standard techniques using blasticidin (Fig. 1b). The functionality of the P protein in the cell line was established by recovering the parental RPV_PPRMFH virus from the plasmid containing a cDNA copy of its genome. This process normally requires transfecting cells with the genome plasmid plus plasmids expressing the N, P and L proteins of the virus. When using the VDS-P cells, the P plasmid could be omitted and live virus recovered (data not shown).

**Fig. 1.**
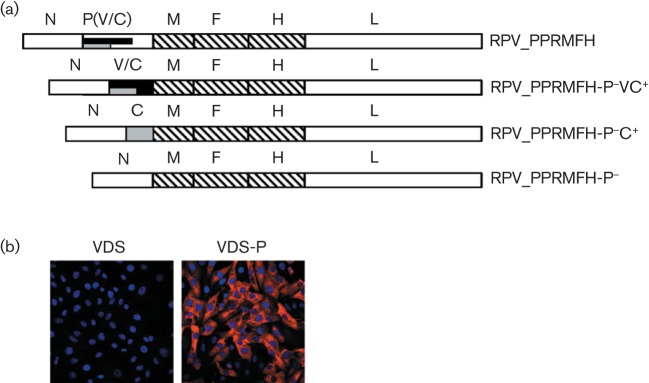
Recombinant viral genomes and helper cell line. (a) Recombinant virus genomes used in these studies. Recombinants were derived from the chimaeric virus RPV_PPRMFH consisting of the core (N/P/L) genes of RPV (plain colour fill) and the shell (M/F/H) genes of PPRV (striped fill); the P gene of this virus expresses P, V and C proteins. The transcription units in the other recombinants mentioned in the text are illustrated. (b) VDS and VDS-P cells were stained with rabbit anti-RPV P antiserum MB18 (Baron *et al.*, 1999) and AlexaFluor568 anti-rabbit IgG (red). Nuclei were counter-stained with DAPI (blue) and the cells were imaged on a Leica laser confocal microscope.

We then attempted the recovery of the various P-deleted virus-like particles (VLPs). Although viruses lacking expression of V and C are viable (Baron & Barrett, 2000; Radecke & Billeter, 1996; Schneider *et al.*, 1997), we could not recover RPV_PPRMFH-P^−^. We were able to observe replication of both P^−^C^+^ and P^−^VC^+^ from the appearance of the viral H protein in the transfected cells. However, only the latter construct grew well enough to spread through the culture and give rise to titratable progeny stock. Multistep growth curves showed that the P^−^VC^+^ VLPs grew reasonably well on VDS-P cells, but nevertheless slower and to lower titre than observed for the P^+^ virus on VDS cells (Fig. 2a); no growth of the P^−^VC^+^ VLPs was seen on normal VDS cells (Fig. 2b). Immunofluorescence showed that much higher levels of viral protein accumulated when cells (with or without extra P protein) were infected with the P^+^ virus than when the P-VLPs were growing on VDS-P cells (Fig. 2b). When we prepared viral antigen for use in the diagnostic cELISA, harvested by the standard methods of freeze–thawing, centrifugation and sonication (Anderson *et al.*, 1990) from VDS-P cells infected with the P^−^VC^+^ VLPs, at the stage when cytopathic effect was advanced, the antigen preparations did not contain sufficient viral protein to react strongly with the mAb, and so could not be used to replace the virus preparations used as standard. These observations suggested that the levels of P protein provided by the cell line are too low to support the normal replication and assembly of the virus genome. Indeed, staining for P protein in cells infected with P^+^ virus showed the accumulation of much higher levels of this protein than seen in the cell line (Fig. 2b). The relatively low level of P protein in the helper cell system may also explain why only the VC^+^ VLP was fully replication competent, despite the evidence that neither V nor C are normally essential. P and V are thought to act as chaperones in the assembly of the nucleocapsid (Spehner *et al.*, 1997; Tober *et al.*, 1998), and it is likely that, at low concentrations of P, the requirement for V increases. Similarly, while the morbillivirus C protein is not essential, its absence does decrease viral replication (Baron & Barrett, 2000; Radecke & Billeter, 1996); combining the deleterious effects of low P levels with those of the absence of C is presumably too much for the viral replication machinery to overcome.

**Fig. 2.**
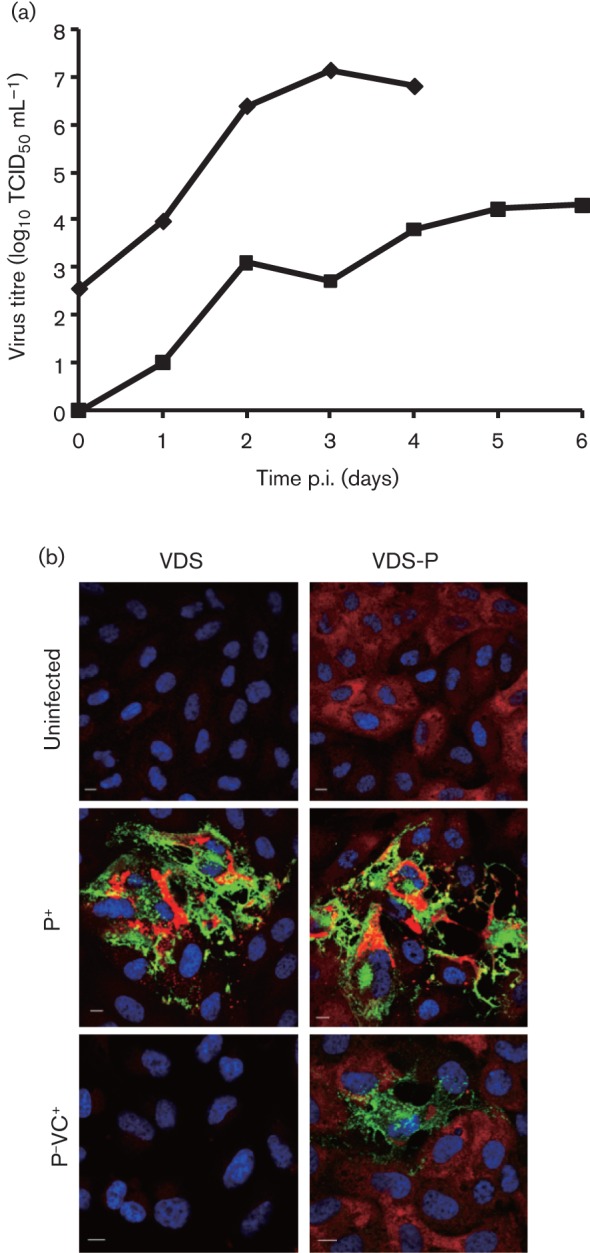
Growth of P-deleted VLPs. (a) Multistep growth of parental (P^+^) virus (⧫) in VDS cells and P-deleted (P^−^VC^+^) VLPs (▪) in VDS-P cells after infection at an m.o.i. of 0.01. Total (cell-associated and supernatant) virus was determined. Note that P^+^ virus growth had killed all the cells at 4 days post-infection (p.i.). (b) Confocal images of VDS or VDS-P cells infected for 24 h with P^+^ virus or P^−^ VLP at an m.o.i. of 0.02, or left uninfected. Cells were fixed and immunostained with rabbit anti-RPV P and mouse anti-PPRV H clone C77 (Anderson *et al.*, 1990) followed by AlexaFluor568 anti-rabbit IgG and AlexaFluor488 anti-mouse IgG. Bars, 10 µm.

These results with the P-deleted morbillivirus contrast with those obtained with a similar strategy used to create a helper cell-dependent filovirus, Ebola virus (Halfmann *et al.*, 2008). In that case, the authors deleted the viral VP30 gene and provided this protein *in trans*. VP30 is not the exact equivalent of the paramyxovirus P protein, being dispensable for viral RNA replication (Mühlberger *et al.*, 1999) and, while required for replication and packaging of the whole virus (Volchkov *et al.*, 2001), the optimal level of VP30 appears to be much lower than for other nucleoprotein components (Mühlberger *et al.*, 1999). Perhaps because of this lower requirement for VP30, the VP30-deleted Ebola virus replicated at a similar rate and to similar titres as the wild-type virus. To use this technique for the production of PPRV antigen, it is apparent that either higher levels of the P protein must be expressed in the cell line or a viral protein selected that is required at lower levels during viral replication. We are currently creating cell lines expressing the PPRV L protein, since all members of the order *Mononegavirales* show a transcription gradient from the promoter-proximal 3′ end of the genome (N gene) to the distal end (L gene), and the L protein is the therefore the least highly expressed of the viral proteins. Only low levels of the L proteins should be required to support normal viral replication. Indeed, overexpression of L can decrease replication (Baron & Barrett, 1997).

Using the P^−^VC^+^ construct, we were able to confirm that the VLPs were completely dependent on the helper cell line. No viral protein was seen in VDS cells even 7 days after infection with P^−^VC^+^ (Fig. 3a). Real-time PCR was able to detect the small amount of genome RNA left on cells during the infection stage (Fig. 3b), and detected a strong production of new viral RNA when VDS-P cells were infected with the P^−^VC^+^ VLPs, but no production of new genomes was observed when normal VDS cells were used, even when infection was allowed to proceed over an extended period (Fig. 3b). Similarly, viral mRNA was only observed when the VLPs were used to infect VDS-P cells, not in VDS cells (Fig. 3c). Putting the VLPs through several blind passages on VDS cells showed no recovery of viral transcription (Fig. 3e, f), just the gradual dilution of the initial amount of viral genome that adhered to the cell, and a failure to produce viral mRNA, showing that there was no trace contaminant of P protein-expressing virus. The gene-deleted virus is therefore completely restricted in its replication to the helper cell line, and such constructs could be considered when production of viral proteins or VLPs has to be carried out for viruses that otherwise require high levels of containment, for growth or transport or both.

**Fig. 3.**
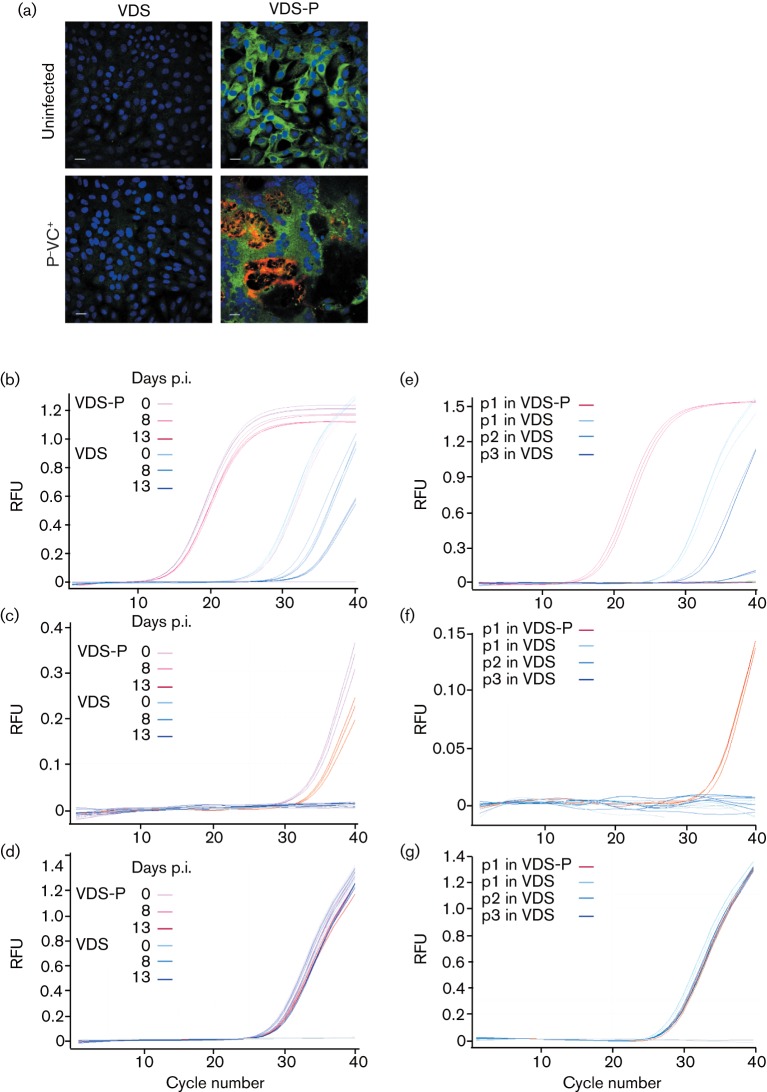
Helper cell dependency of VLP growth. (a) VDS and VDS-P cells were infected with P^−^VC^+^ VLPs at an m.o.i. of 0.01 and fixed at 7 days p.i. The cells were stained as described in Fig. 2(b), except that the secondary antibodies were AlexaFluor568 anti-mouse IgG and AlexaFluor488 anti-rabbit IgG. Bars, 25 µm. (b–d) VDS and VDS-P cells were infected with P^−^VC^+^ VLPs at an m.o.i. of 0.005. Cells were harvested immediately, and at 8 and 13 days p.i. and total RNA purified using the Qiagen RNeasy kit. cDNA was reverse transcribed from 100 ng RNA using either (b) random primers or (c, d) the poly(A)-specific primer (T)_16_VN; the relative amount of viral RNA (genome+mRNA) (b) or viral mRNA (c) was determined using RPV N gene-specific primers (sequence provided on request). The mRNA for ribosomal protein L13A (d) was used as an internal control. (e–g) The P^−^VC^+^ VLPs were blind passaged three times (p1, p2 and p3) in VDS cells for 7 days each passage, and total cellular RNA prepared after each passage. The relative amount of (e) total viral RNA, (f) viral mRNA and (g) internal control L13A mRNA was determined in each RNA preparation as described above. RNA from the first passage of the VLPs in VDS-P cells was used as a positive control. RFU, Relative fluorescence units.
